# A population genomic characterization of copy number variation in the opportunistic fungal pathogen *Aspergillus fumigatus*

**DOI:** 10.1371/journal.pone.0201611

**Published:** 2018-08-02

**Authors:** Shu Zhao, John G. Gibbons

**Affiliations:** Biology Department, Clark University, Worcester, Massachusetts, United States of America; Brigham Young University, UNITED STATES

## Abstract

*Aspergillus fumigatus* is a potentially deadly opportunistic fungal pathogen. Molecular studies have shaped our understanding of the genes, proteins, and molecules that contribute to *A*. *fumigatus* pathogenicity, but few studies have characterized genome-wide patterns of genetic variation at the population level. Of *A*. *fumigatus* genomic studies to-date, most focus mainly on single nucleotide polymorphisms and large structural variants, while overlooking the contribution of copy number variation (CNV). CNV is a class of small structural variation defined as loci that vary in their number of copies between individuals due to duplication, gain, or deletion. CNV can influence phenotype, including fungal virulence. In the present study, we characterized the population genomic patterns of CNV in a diverse collection of 71 *A*. *fumigatus* isolates using publicly available sequencing data. We used genome-wide single nucleotide polymorphisms to infer the population structure of these isolates and identified three populations consisting of at least 8 isolates. We then computationally predicted genome-wide CNV profiles for each isolate and conducted analyses at the species-, population-, and individual levels. Our results suggest that CNV contributes to genetic variation in *A*. *fumigatus*, with ~10% of the genome being CN variable. Our analysis indicates that CNV is non-randomly distributed across the *A*. *fumigatus* genome, and is overrepresented in subtelomeric regions. Analysis of gene ontology categories in genes that overlapped CN variants revealed an enrichment of genes related to transposable element and secondary metabolism functions. We further identified 72 loci containing 33 genes that showed divergent copy number profiles between the three *A*. *fumigatus* populations. Many of these genes encode proteins that interact with the cell surface or are involved in pathogenicity. Our results suggest that CNV is an important source of genetic variation that could account for some of the phenotypic differences between *A*. *fumigatus* populations and isolates.

## Introduction

*Aspergillus fumigatus* is a ubiquitous, saprophytic mold found in soil, compost, and other organic matter, and plays an important ecological role as a decomposer [1, 2]. This species is also an opportunistic human pathogen and is responsible for the greatest number of deaths and the second highest number of infections of any fungal species [3]. It is estimated that *A*. *fumigatus* infection in immunocompromised individuals results in 100,000 deaths annually [4]. *A*. *fumigatus* harbors several strategies conducive to the pathogenic lifestyle. The small size of the conidia and layer of hydrophobic proteins covering the conidia permits evasion of mucociliar clearance, and mask the antigenic carbohydrate β(1,3)-glucan, which alveolar macrophages use for recognition, respectively [1]. *A*. *fumigatus* also grows optimally at 37°C, which, coincidentally, is the internal temperature of the human body [5], and produces an arsenal of molecules used to degrade host tissue, import nutrients, and counteract host defenses [1, 2, 6].

High-throughput sequencing combined with comparative and population genomics is a powerful tool for identifying genes or genetic variants associated with phenotypes, including components of *A*. *fumigatus* pathogenicity. The utility and power of this approach was first exhibited in a study by Camps *et al*. [7], in which the resequencing and comparison of serial isolates collected from a patient before and after prolonged azole therapy revealed a novel mutation in the *hapE* gene that conferred azole resistance. Whole-genome sequencing of patient derived serial isolates resulted in the identification of several nonsynonymous mutations, a 38.5 Kb deletion containing 11 genes, and the presence of an isolate with the azole resistant *cyp51A* mutation [8]. In one of the most extensive *A*. *fumigatus* population genomic studies to date, Abdolrasouli *et al*. [9] resequenced the genomes of 24 *A*. *fumigatus* isolates to characterize genetic variants associated with azole resistance. This study confirmed that the TR34/L98H mutation in the cyp51A gene is the sole mechanism responsible for azole resistance in the analyzed isolates and also provided evidence for recombination, including in those isolates with the TR34/L98H mutation.

The majority of the aforementioned genomic studies analyzed single nucleotide polymorphisms (SNPs) or large scale structural variants while playing little or no attention to copy number variation (CNV). CNV is a type of segregating variation that is defined as fragments of DNA that are present at variable copy number (CN) in comparison with a reference genome [10]. CNV mutation rates are often higher than those of SNPs [11, 12], and are the result of several mutational processes including non-allelic homologous recombination, non-homologous end-joining, retrotransposition, and fork stalling and template switching [13–16]. CNV can affect phenotype by directly altering gene function through gene interruption, or gene fusion, or by modifying gene expression through gene dosage, regulatory element dosage, and position effect [17]. In fungi, gene CNV has been associated with phenotypic variation and adaptation [18]. For example, population genomic analyses revealed widespread CNV in fermentation-related genes in *Saccharomyces cerevisiae* wine strains [19], higher α-amylase gene expression in isolates of *Aspergillus oryzae* and *Aspergillus flavus* with greater α-amylase CN [20], and differences in pathogenicity-related gene CN between closely related, but phenotypically distinct, populations of *Cryptococcus gattii* [21]. A recent population genomic analysis of *A*. *fumigatus* secondary metabolite encoding gene clusters revealed widespread gene CNV which likely contributes to phenotypic variation [22].

Despite the importance of CNV as a source of genetic and phenotypic variation, no study to date has characterized the genome-wide population patterns of CNV in *A*. *fumigatus*. In the present study we analyzed publicly available whole-genome Illumina sequence data from 71 *A*. *fumigatus* isolates [9, 23–25]. We first identified three genetic populations consisting of at least 8 individuals using a panel of high-resolution SNPs. We then performed multiple analyses to characterize the abundance, localization, variation, and functional associations of CNVs at the species-, population-, and individual-levels.

## Materials and methods

### Data-mining and sequence processing

Whole genome paired-end Illumina sequence data for 71 *A*. *fumigatus* isolates [9, 23–25] was downloaded from the NCBI Sequence Read Archive [26] using the SRA toolkit (S1 Table). We implemented a similar data processing pipeline described previously [21]. Briefly, identical paired-end sequence reads were collapsed using tally, with the parameters “—with-quality” and “—pair-by-offset” [27]. Next, trim_galore (http://www.bioinformatics.babraham.ac.uk/projects/trim_galore/) was used to remove residual adapter sequences, and to trim reads at bases where quality scores were below Q30. Trimmed reads shorter than 50 bp were removed. These read sets were then mapped to the *A*. *fumigatus* Af293 reference genome [28] using the “sensitive” pre-set parameters in bowtie2 [29]. SAM alignment files were converted into sorted BAM format using the *view* and *sort* functions in samtools [30]. The samtools *depth* function was then used to estimate average coverage for each of the 71 samples. To avoid the bias introduced by varying sequencing depths across samples, seqtk (https://github.com/lh3/seqtk) was used to randomly subsample reads such that each sample had a genome-wide average coverage of 10X. These deduplicated, quality and adapter trimmed, 10X coverage read sets were mapped against the *A*. *fumigatus* Af293 reference genome [28] and used in subsequent SNP and CNV analysis.

### Identifying *A*. *fumigatus* populations

We performed three analyses to infer the population structure of the 71 *A*. *fumigatus* isolates. SNP sites for each sample were conservatively predicted using VarScan v2.3.9 with the parameters “—min-var-freq 1” and “—min-coverage 8” [31]. Consensus genotypes from polymorphic sites were extracted for each sample. Sites harboring more than 5% missing or ambiguous data were removed. This process resulted in 35,120 variant sites. We also subsampled polymorphic sites to minimize physical linkage between markers, resulting in 859 sites separated by an average distance of ~33 Kb.

We first performed population structure analysis with the subsampled set of 859 variant sites using the Structure v2.3.4 while implementing the “admixture” ancestry model, and the “allele frequencies are correlated among populations” frequency model [32]. We ran 15 replicates with a burn-in length of 100,000, and a Markov Chain Monte Carlo (MCMC) of 200,000 generations for *K* = 1–15, where *K* indicates the number of genetic clusters or populations. Δ*K*, a measurement of the rate of change in the average log probability between successive *K* values, was calculated using Structure Harvester in order to predict the optimal number of populations [33, 34]. We additionally used admixture with the subsampled set of 859 variant sties, and the full set of 35,120 variant sites to compare individual population assignments [35]. admixture was run for *K* = 1–15. Cross validation error was calculated for each *K* value with the lowest values corresponding to good predictors of *K*. Lastly, we constructed a Neighbor-Net phylogenetic network using the set of 859 variant sites with SplitsTree V4.14.4 with 1,000 bootstrap replicates [36].

### Copy number variation analysis

We used the read depth based approach implemented in control-FREEC to estimate integer CN for each non-overlapping 500 bp window throughout the genome [37]. The following parameters were used: window = 500, telocentromeric = 0, minExpectedGC = 0.33, and maxExpectedGC = 0.63. Heatmaps of CNV patterns were illustrated using the R package ComplexHeatmap [38].

We calculated two measurements of CNV diversity. First, we calculated the Polymorphic Index Content (PIC) across all samples, and within each population [39, 40]. PIC is a useful measurement for the identification of diverse CN variable loci [19], and ranges from 0 (no CNV is present) to 1 (all alleles are unique). PIC was calculated as follows:

$$PIC=1-\sum_{a}^{z}i^{2}$$

where *i*^*2*^ is the squared frequency of *a* to *z* CN values at a particular 500 bp window. PIC values in the upper 99th percentile of all samples, corresponding to values greater than 0.82, were considered significant within each population.

We calculated V_ST_ to identify divergent CNV profiles between populations [12]. V_ST_ is conceptually similar to F_ST_ and varies from 0 (no difference in CN allele frequencies between populations) to 1 (completely differentiation of CN allele frequencies between populations). V_ST_ was calculated as follows:

VST=Vtotal−(Vpop1×Npop1+Vpop2×Npop2+Vpop3×Npop3)NtotalVtotal

where V_total_ is total variance, V_popx_ is the CN variance for each respective population, N_VGIIx_ is the sample size for each respective population, and N_total_ is the total sample size. We considered V_ST_ values in the upper 99th percentile, corresponding to values greater than 0.68, as significantly differentiated between populations.

### Genomic localization of copy number variable genes

To test whether CN variants were disproportionately represented in subtolemeric regions, we compared the number of observed CN variants that partially or entirely overlapped the subtelomeric regions to the number expected if CN variants were randomly distributed across the genomes. The proportion of observed vs. expected was assessed using a chi-square goodness of fit test. This analysis was conducted independently for each chromosome, and for the entire genome. Subtelomeric regions were defined as the 400 kb region preceding the telomere end. We ran nine independent tests (1 test per chromosome plus 1 test for the entire genome), and thus implemented a Bonferroni multiple test-corrected p-value cutoff of 0.0056 (p-value cut-off = 0.05 / 9 tests = 0.0056).

### Gene ontology enrichment

We performed Gene Ontology (GO) enrichment across all samples, and within each population for genes in which CN variants partially overlapped, and for genes in which gene entirely overlapped. All GO enrichment analysis was performed in Fungifun2 [41] using the *A*. *fumigatus* Af293 annotation from AspGD [42].

## Results

### Population structure analysis

**Fig 1:**
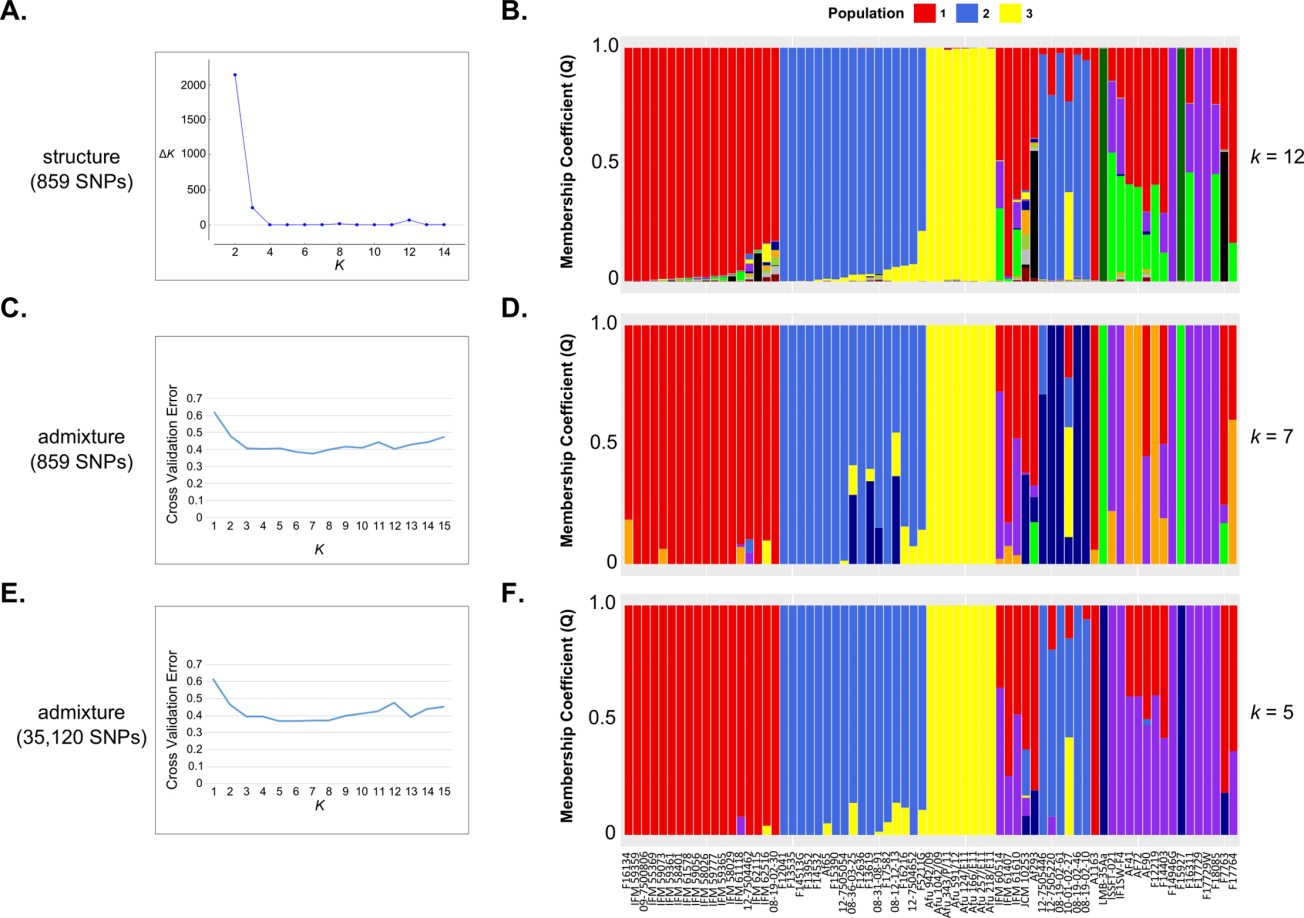
The evolutionary relationship of 71 *Aspergillus fumigatus* isolates. (A) Structure based estimate of the optimal predicted population number (*K*) by the Δ *K* statistic using 859 SNPs. (B) Structure based population assignment for the 71 *A*. *fumigatus* isolates for *K* = 12. Columns correspond to different individuals (X-axis), while membership coefficient is depicted on the Y-axis. Admixture based estimate of the optimal predicted population number (*K*) by the cross validation error method using 859 SNPs (C) and 35,120 SNPs (E). Admixture based population assignment for the 71 *A*. *fumigatus* isolates for *K* = 7 with 859 SNPs (D) and *K* = 5 with 35,120 SNPs (F). Columns correspond to different individuals (X-axis), while membership coefficient is depicted on the Y-axis. Isolates colored with red, blue, and yellow correspond to populations 1, 2, and 3.

Our aim was to analyze patterns of CNV at the species, population, and individual levels. Thus, we first determined the evolutionary relationships and population structure of the 71 *A*. *fumigatus* isolates. Using a collection of 859 SNPs distributed across the genome, we used Structure to predict population structure (Fig 1A and 1B) [32, 43]. We calculated Δ*K* for each *K* value [33] and this analysis suggested that *K* = 2, *K* = 3 and *K* = 12 represent the best predictors of population number (Fig 1A). To better understand why *K* = 2 and *K* = 3 gave the strongest signal, we constructed a Neighbor-Net phylogenetic network of the 71 *A*. *fumigatus* isolates (S1 Fig). This analysis revealed that two closely related isolates (LMB-3Aa, and F15927) were highly divergent to all other isolates (Fig 1B) [44]. When *K* = 3 these isolates also formed a single population. When *K* = 12, we find strong agreement in population assignment between structure, admixture, and the phylogenetic network (Fig 1 and S1 Fig). Population structure analysis was further investigated using admixture [35] with the subsampled set of 859 variant sites (Fig 1C and 1D), and the full set of 35,120 variant sites (Fig 1E and 1F). The best predictor of population number was 7 and 5 when the subsampled set of 859 variant sites, and the full set of 35,120 variant sites were used, respectively (Fig 1C and 1E).

Together, these analyses (structure with 859 variant sites, admixture with 859 variant sites, admixture with 35,120 variant sites, and SplitsTree with 859 variant sites) resulted in a concordant set of individuals falling into three populations with a sample size ≥ 8 (Fig 1 and S1 Fig). Population 1 consists mainly of clinical isolates from Japan [23], but also two clinical isolates from the United Kingdom, and one environmental isolate from the Netherlands [9]. Population 2 consists primarily of clinical isolates with azole resistance from the United Kingdom and the Netherlands [9]. Population 3 consists of clinical isolates with azole resistance from India [9]. The remaining samples are composed of both clinical and environmental isolates that have both azole susceptibility and azole resistance from Asia, Europe, North America, South America and the International Space Station [9, 23, 25, 44]. Our subsequent analyses of CNVs at the species-level are conducted with all 71 isolates, while the population-level analyses consisted of the 43 isolates from populations 1, 2, and 3 (Fig 1 and S1 Table).

### Characterizing copy number variation at the species-level

**Fig 2:**
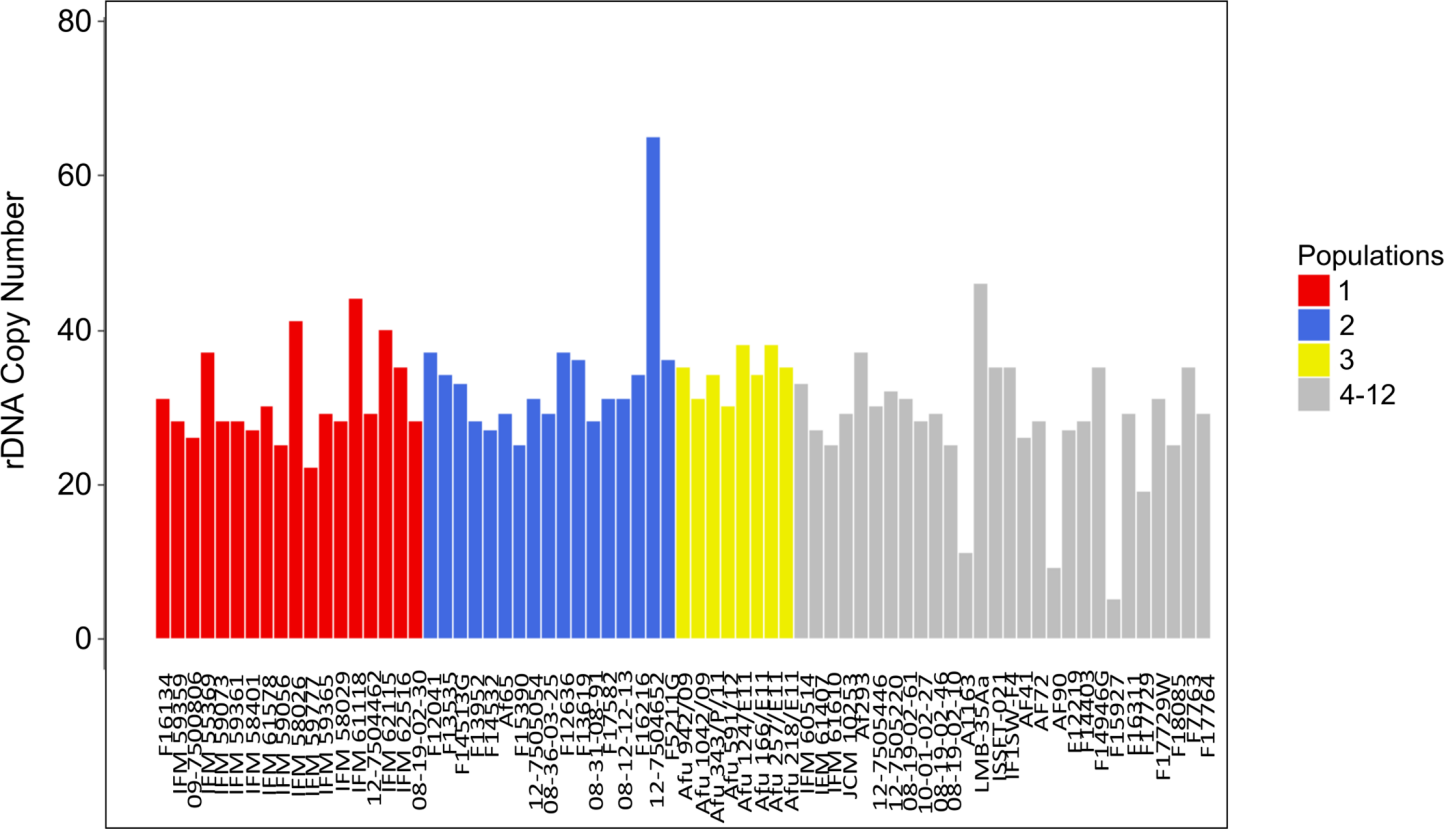
Copy number of the ribosomal DNA (rDNA) locus across the 71 *A*. *fumigatus* isolates. Columns represent individuals (X-axis). rDNA copy number is depicted on the Y-axis. Isolates from population 1, population 2, population 3, and populations 4–12 are represented in red, blue, yellow, and grey, respectively.

We generated CNV profiles for each non-overlapping 500 bp window throughout whole genome of the 71 *A*. *fumigatus* isolates using control-FREEC [37] (S1 File). To assess our computational CNV prediction pipeline, we first examined the CN of the ribosomal DNA (rDNA) encoding locus and compared these results to previous studies that used quantitative PCR and digital droplet PCR to quantify rDNA CN [45, 46]. The average rDNA CN of the 71 *A*. *fumigatus* isolates ranged from 5 to 65, with an average of 30.72 and median of 30 (Fig 2). We are encouraged by our *in silico* results as they are within the range of experimentally determined *A*. *fumigatus* rDNA CN estimates [45, 46].

**Fig 3:**
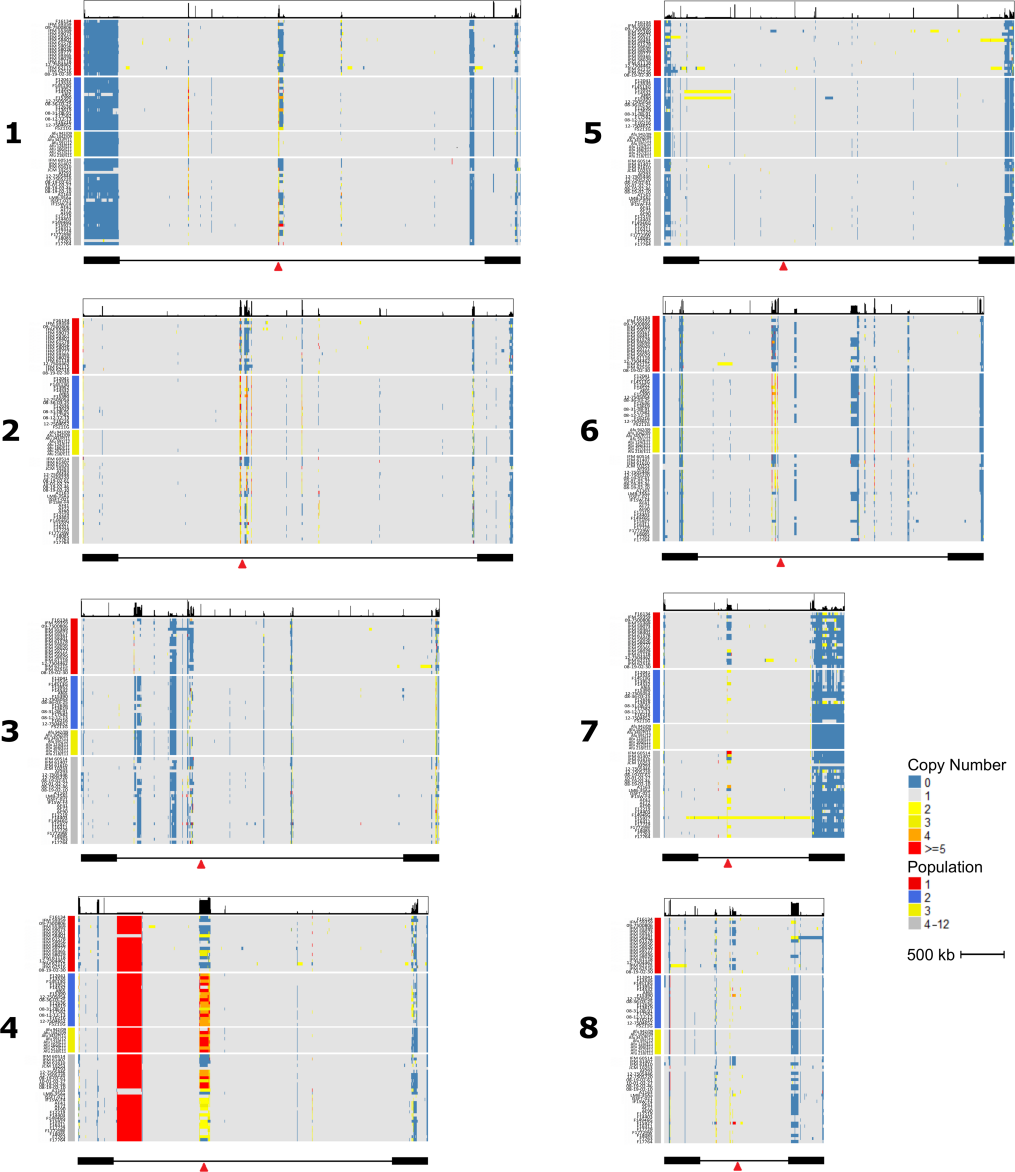
Genome-wide copy number variation profiles across the 71 *A*. *fumigatus* isolates. Heatmaps depicting copy number are represented for each chromosome, with blue, grey, light yellow, dark yellow, orange, and red representing copy number of 0, 1, 2, 3, 4, and > = 5, respectively. Colors of copy numbers greater than 2 are indicated in the legend. Rows represent individuals with colors of red, blue, yellow, and grey depicting populations 1, 2, 3, and 4–12, respectively. The thick black bars under each heatmap represent subtelomeric regions to the end of chromosome and the red arrow indicates the putative location of centromere.

**Fig 4:**
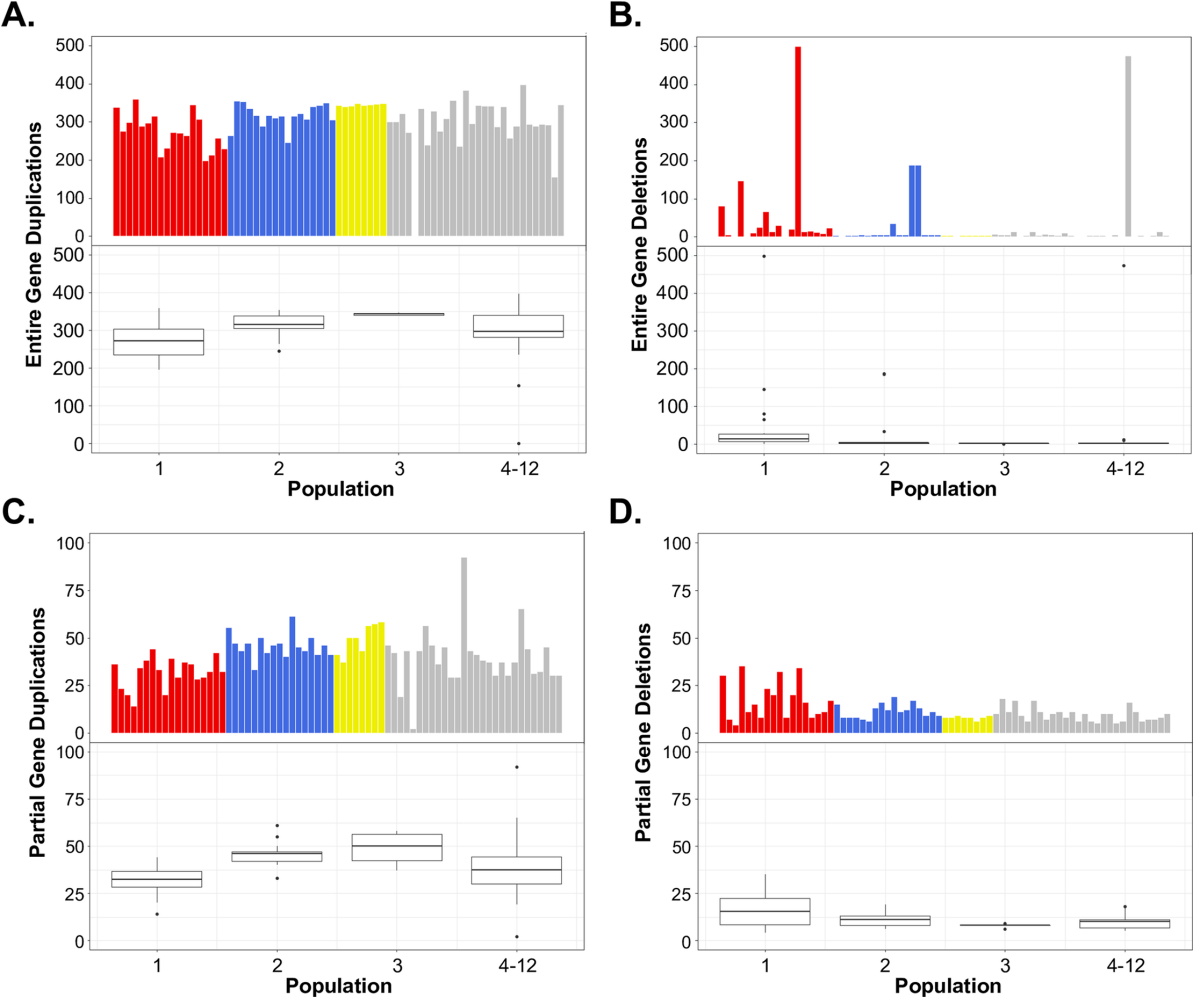
**The number of entire (A), and partial (B) gene absences and entire (C) and partial (D) gene gains in each of the 71 *A*. *fumigatus* isolates.** Individuals from populations 1, 2, 3, and 4–12 are represented in red, blue, yellow, and grey, respectively. The length of the box shows the interquartile range, the top of the upper whisker shows the largest data point less than the 3^rd^quartile + 1.5*(interquartile range), the bottom of the lower whisker represents the smallest data point less than the 1^st^ quartile—1.5*(interquartile range).

On average, 7.88% of the genome (~2.31 Mb) is CN variable, with 5.94% (1.74 Mb) and 1.94% (0.57 Mb) deriving from absences and duplications, respectively (Fig 3). We choose to refer to CN of 0 as an “absence” rather than a deletion because the absence of a locus in an analyzed genome could be the result of a gain in the reference genome and not solely a deletion in the analyzed genomes. The average number of absences and gains per isolate is 89 and 76, respectively. Our collection of analyzed isolates included AF293 (Fig 1), which also served as our reference genome. We observed very few CNVs in the AF293 isolate further reinforcing the accuracy of our CNV prediction (Fig 3). We examined the number of CNVs that overlapped annotated genes across all isolates, and discovered substantial variation. In at least one of the 71 isolates, 433 and 922 genes overlapped partially and completely with absences, respectively. Because we observed a wide-range in gene gains across isolates that were likely the result of rare large segmental duplications (Figs 3 and 4), we separated the 71 isolates into two groups according to the number of gene gains. Isolates with the number of gene gain events greater than the 3^rd^ quartile + 1.5*(interquartile range) were considered as group 2 isolates, while all other isolates were considered as group 1. Group 1 included 62 isolates that harbored fewer than 27 duplicated genes, while group 2 included the remaining 9 isolates that possessed greater 27 gene duplications (Fig 4). In the duplicated regions, we found 126 and 1,310 entire gene gains in group 1 and group 2, respectively, with 75 genes shared between the groups. When analyzing patterns of gene CNV that were present in at least 2 isolates, we found that more gene absences are shared between isolates (60.2% of absent genes) than gene duplications (26.89% of duplicated genes) (Fisher’s exact test; *p*-value = 2.2e-16).

**Fig 5:**
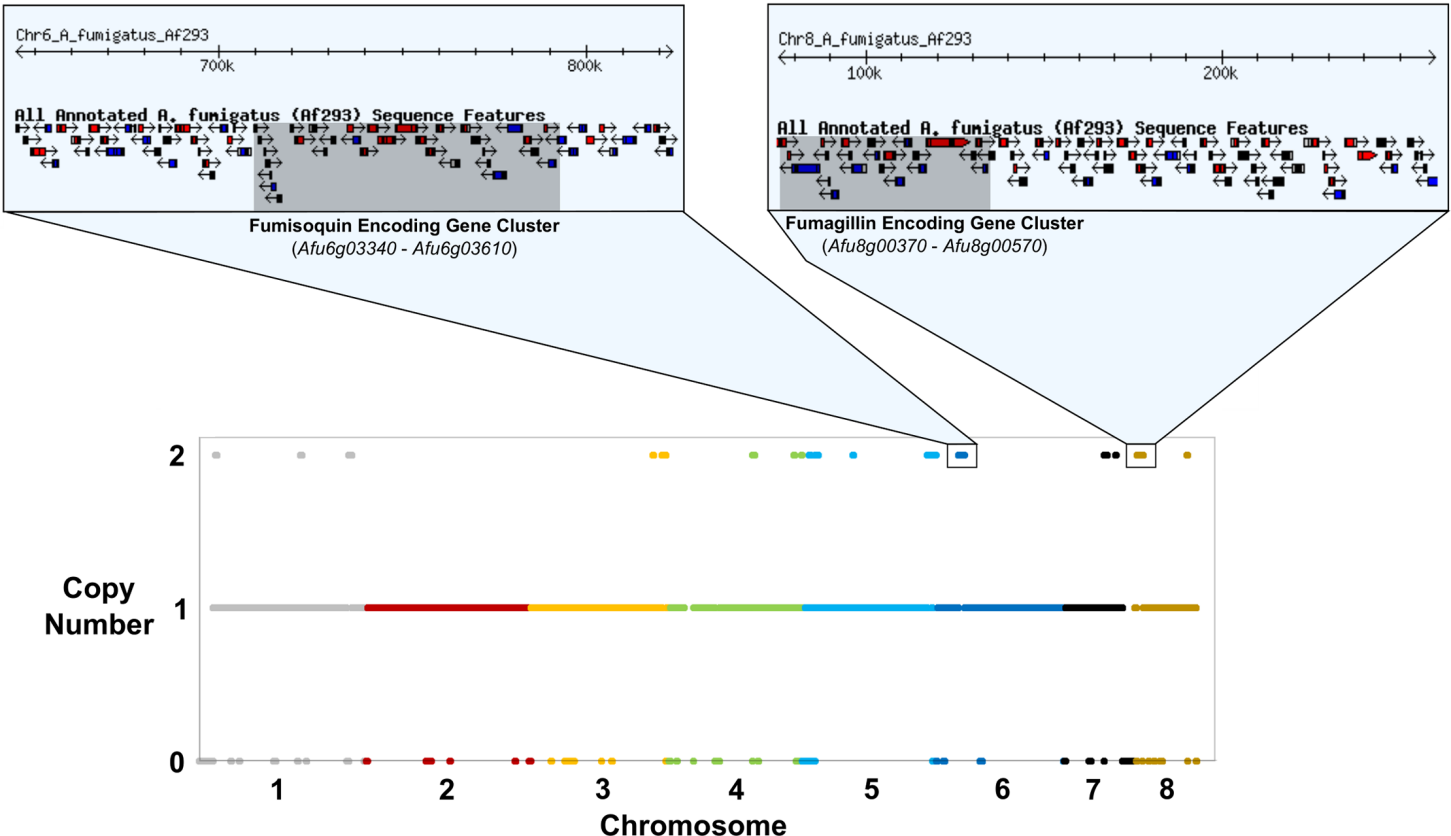
Large segmental duplications overlapping genes in isolate Afum IFM 62115. The lower panel depicts copy number (Y-axis) of each 500 bp non-overlapping window in the genome (X-axis). Each color represents a chromosome. For clarity, copy numbers greater than two are not shown. The outlined loci in chromosomes 6, and 8 represent segmental duplications larger than 160 kb. The upper panel depicts the gene content of these duplicated regions. The shaded box on chromosome 6 encompasses the fumisoquin encoding gene cluster (*Afu6g03340*—*Afu6g03610*), while the shaded box on chromosome 8 encompasses the fumagillin encoding gene cluster (*Afu8g00370*—*Afu8g00570*).

Among 71 isolates, the size of duplication events ranged from 500 bp to 537 kb with an average of 7.5 Kb and a median of 1.5 Kb. As noted, 9 of the *A*. *fumigatus* isolates (group 2) contained relatively large segmental duplications (Figs 3 and 4). The cumulative size of duplications was significantly greater in group 2 isolates (760.6 Kb) compared to group 1 isolates (344.5 Kb) (Students T-test; *p*-value = 0.036). Among the 9 group 2 isolates, we observed 43 segmental duplications events larger than 40 kb, covering between 11 and 474 genes. For example, isolate Afum IFM 62115 [23] contained two independent duplications larger than 160 kb that entirely overlapped the fumisoquin and fumagillin encoding secondary metabolic gene clusters (Fig 5) [47].

We observed an overrepresentation of CN variants in subtelomeric regions (defined as 400 Kb from chromosome end [24, 48] (Fig 3). On average, 68.6% of CNV absences fell within subtelomeric regions compared to a probability of 2.5% if absences occurred randomly throughout the genome (Fisher’s exact test; *p*-value < 2.2e-16). This trend is consistent across individual chromosomes (Fisher’s exact test; chromosomes 1, 2, 4, 5, 6, 7, and 8: *p*-value < 2.2e-16, and chromosome 3: *p*-value = 1.84e-5).

We performed Gene Ontology enrichment analysis of these CN variable genes to better understand their broad functional associations. For the collection of entirely absent genes, a cohesive set of overrepresented GO terms were associated with the presence of transposable elements. These terms include RNA-directed DNA polymerase activity (GO:0003964; p-value = 2.71e-20), RNA-dependent DNA replication (GO:0006278; 1.93e-19), RNA-DNA hybrid ribonuclease activity (GO:0004523; 1.53e-13), and DNA integration (GO:0015074; p-value = 0.03) (Table 1). For entirely duplicated genes, several GO terms were significantly overrepresented: fumagillin biosynthetic process (GO:1902086; p-value = 1.9526e-13), oxidoreductase activity, acting on paired donors, with incorporation or reduction of molecular oxygen (GO:0016705; p-value = 0.019), electron carrier activity (GO:0009055; p-value = 0.019), oxidation-reduction process (GO:0055114, p-value = 0.03), secondary metabolic process (GO:0019748; p-value = 0.04), and iron ion binding (GO:0005506; p-value = 0.04) (Table 2).

**Table 1:** Gene ontology enrichment for entirely absent genes.

GO ID	GO category name	GO namespace	Adjusted *p*-value	Number of genes
GO:0005575	cellular component	CC	4.2377e-43	612
GO:0003964	RNA-directed DNA polymerase activity	MF	2.7091e-20	21
GO:0006278	RNA-dependent DNA replication	BP	1.9251e-19	21
GO:0008150	biological process	BP	1.3398e-16	473
GO:0004523	RNA-DNA hybrid ribonuclease activity	MF	1.5308e-13	16
GO:0003677	DNA binding	MF	0.000022699	57
GO:0003723	RNA binding	MF	0.0057492	23
GO:0003674	molecular function	MF	0.011892	406
GO:0015074	DNA integration	BP	0.029265	3

BP = biological process

CC = cellular component

MF = molecular function

**Table 2:** Gene ontology enrichment for entirely duplicated genes.

GO ID	GO category name	GO namespace	Adjusted *p*-value	Number of genes
GO:1902086	fumagillin biosynthetic process	BP	1.9526e-13	15
GO:0005575	cellular component	CC	2.4798e-12	603
GO:0016705	oxidoreductase activity, acting on paired donors, with incorporation or reduction of molecular oxygen	MF	0.019211	18
GO:0009055	electron carrier activity	MF	0.019211	23
GO:0055114	oxidation-reduction process	BP	0.02849	80
GO:0019748	secondary metabolic process	BP	0.041801	13
GO:0005506	iron ion binding	MF	0.041801	24

BP = biological process

CC = cellular component

MF = molecular function

**Fig 6:**
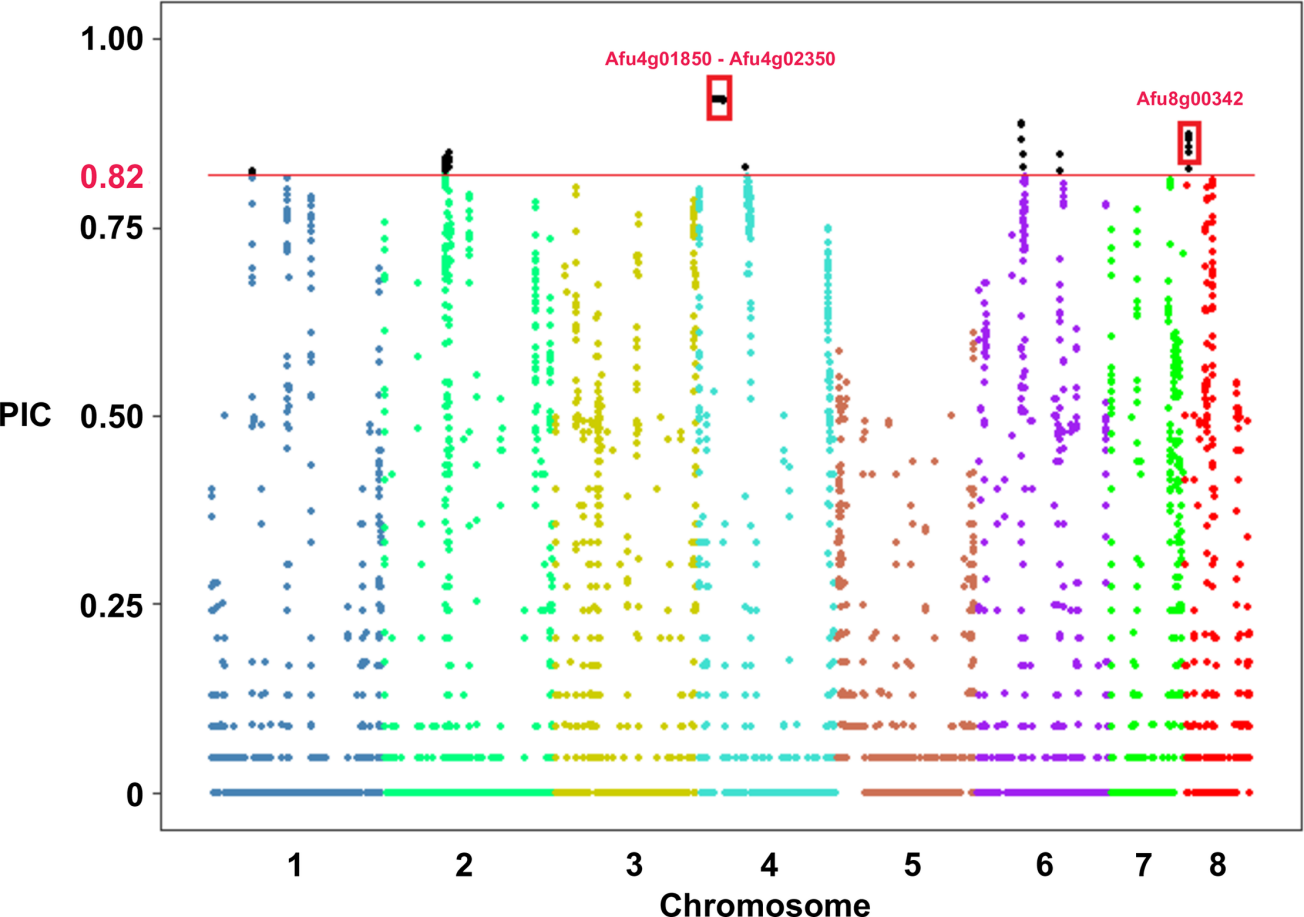
Highly polymorphic copy number variants in *A*. *fumigatus*. Manhattan plot of the Polymorphic Index Content (PIC) value (Y-axis) for each 500 bp non-overlapping window in the genome (x-axis) across the 43 samples from populations 1, 2, and 3. The horizontal red line represents the upper 99% percentile of PIC values. The loci outlined in red boxes on chromosomes 4 and 8 represent the regions that overlapped entire genes.

We also investigated the diversity of CNV loci using the Polymorphic Index Content (PIC) measurement. PIC has been used to estimate diversity of microsatellites, restriction fragment length polymorphisms, and CNVs [19, 49]. We calculated PIC for each non-overlapping 500 bp region of the genome. The average PIC value for regions of the genome with CNV in at least 1 isolate is 0.04, suggesting most regions of the genome lack CNV diversity. We identified 613 windows in the top 1% of PIC values (≥ 0.82) (Fig 6), representing 25 distinct loci. Four and one of these high diversity CNV regions partially and completely overlapped genes, respectively. Three of the four genes mapped to the rDNA locus, while the remaining gene (*Afu8g00342*) plays a predicted role in the Pseurotin A secondary metabolite encoding gene cluster [50].

### Divergent copy number profiles between *A*. *fumigatus* populations

**Fig 7:**
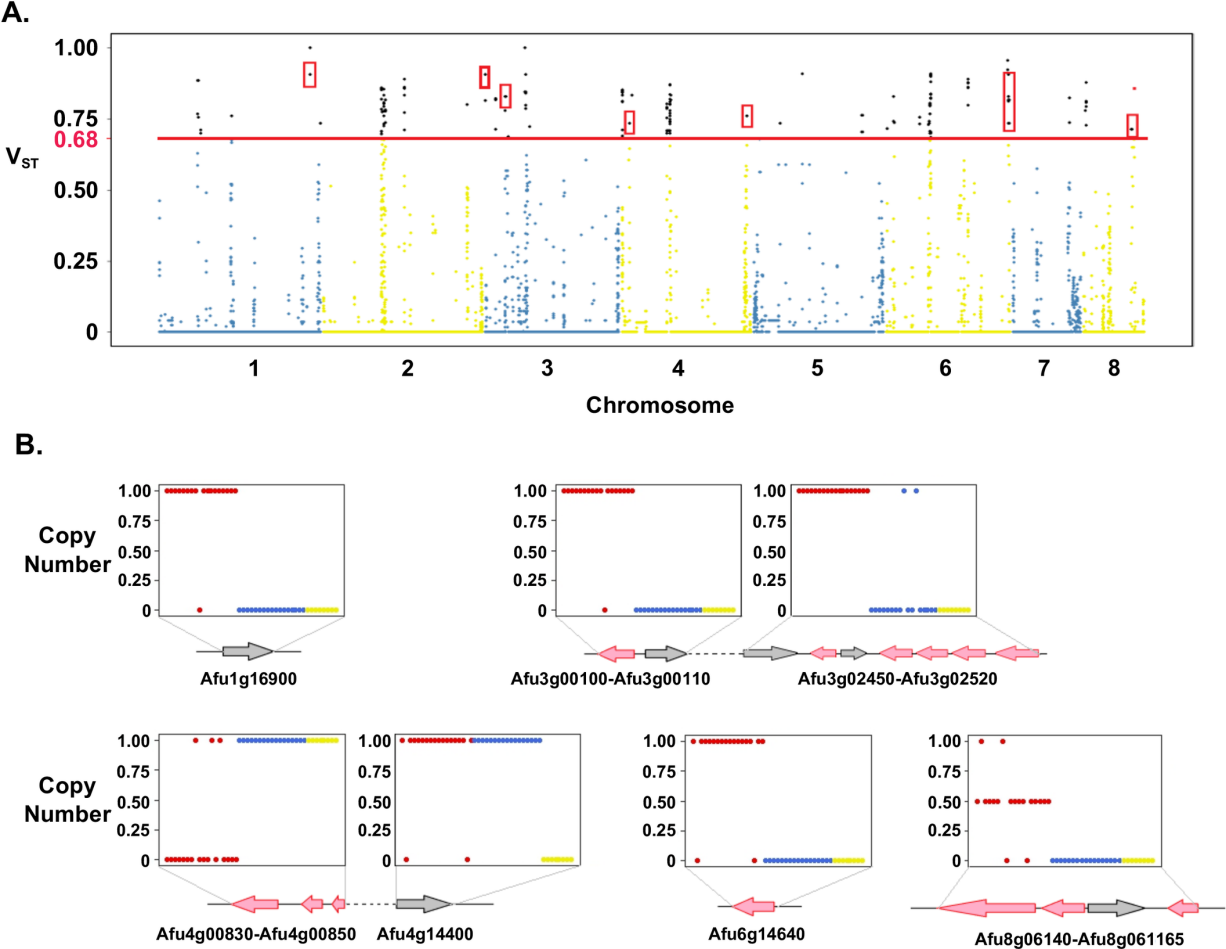
Loci with highly differentiated copy number between *A*. *fumigatus* populations. (A) V_ST_ values (y-axis) for every non-overlapping 500 bp window across the 8 chromosomes (x-axis). The horizontal red line represents the upper 99% percentile of V_ST_ values. Loci encompassing genes are outlined in red boxes. (B) High V_ST_ regions that entirely overlap genes. For each plot, the Y axis represents copy number, while X-axis represents individuals from populations 1 (red), 2 (blue), and 3 (yellow). Arrows represent genes, and their orientations corresponds to the direction of transcription.

Population processes, including natural selection, can shape CN patterns between populations [19, 51]. To identify loci differentiated by CN between populations, we calculated V_ST_ at each 500 bp window between populations 1, 2, and 3. V_ST_ is conceptually derived from F_ST_ [52, 53], and ranges from 0 to 1, with a value of 0 representing complete allelic sharing between populations, and a value of 1 representing fixed allelic differences between populations. The average V_ST_ value for regions of the genome with CNV in at least 1 isolate is 0.022, suggesting the majority of the genome is not differentiated by population. We considered the upper 99% of V_ST_ values as significant, representing a cutoff of V_ST_ = 0.68. In total, we identified 545 divergent CN variable windows, comprising 72 distinct non-overlapping loci. These high V_ST_ regions contained 33 genes, 19 and 14 of which were completely and partially overlapped by CN variants, respectively (Fig 7 and Table 3). Several proteins encoded by these high V_ST_ genes localize to or interact with the cell membrane including transmembrane transporters (*Afu3g02520*, *Afu4g00830*, *Afu5g12720*, and *Afu6g14640*), and kinases (*Afu3g02460*, *Afu3g02500*, and *Afu8g06140*) (Table 3). Other genes present in the high V_ST_ regions were associated with putative pathogenicity functions, such as oxidation reduction (*Afu3g00100*, and *Afu3g00110*) and hydrolase activity (*Afu4g01070*) [1]. In addition, we identified a high V_ST_ locus containing 7 genes that are part of a highly variable secondary metabolism gene cluster [24]. This locus contains at least 6 distinct and unrelated secondary metabolism gene cluster “alleles”[54].

**Table 3:** Genes overlapping high V_ST_ regions of genome.

Gene ID	Overlap Type	Gene Ontology Molecular Function	Gene Ontology Biological Process	Gene Ontology Cellular Component
*Afu1g04400*	Partial	structural constituent of ribosome	mitochondrial translation	cytosol; mitochondrial small ribosomal subunit; nucleus
*Afu1g16900*	Complete	N/A	N/A	N/A
*Afu3g00100*	Complete	oxidoreductase activity	oxidation-reduction process	N/A
*Afu3g00110*	Complete	succinate-semialdehyde dehydrogenase [NAD(P)+] activity	gamma-aminobutyric acid catabolic process; oxidation-reduction process	N/A
*Afu3g02450*	Complete	N/A	N/A	N/A
*Afu3g02455*	Complete	N/A	N/A	N/A
*Afu3g02460*	Complete	ATP binding; protein tyrosine kinase activity	protein phosphorylation	N/A
*Afu3g02470*	Complete	amidase activity; carbon-nitrogen ligase activity, with glutamine as amido-N-donor	N/A	N/A
*Afu3g02480*	Complete	RNA polymerase II transcription factor activity, sequence-specific DNA binding; zinc ion binding	regulation of transcription, DNA-templated	nucleus
*Afu3g02500*	Complete	ATP binding; protein tyrosine kinase activity	protein phosphorylation	N/A
*Afu3g02520*	Complete	N/A	transmembrane transport	integral component of membrane
*Afu3g04270*	Partial	N/A	N/A	N/A
*Afu4g00830*	Complete	dipeptide transporter activity; tripeptide transporter activity	dipeptide transport; tripeptide transport	membrane
*Afu4g00810*	Partial	N/A	N/A	N/A
*Afu4g00840*	Complete	N/A	N/A	N/A
*Afu4g00850*	Complete	N/A	N/A	N/A
*Afu4g01070*	Partial	hydrolase activity	pathogenesis	cell surface; cell wall-bounded periplasmic space; extracellular region
*Afu4g14400*	Complete	N/A	N/A	N/A
*Afu4g14410*	Partial	N/A	N/A	N/A
*Afu5g02980*	Partial	DNA binding	N/A	N/A
*Afu5g06180*	Partial	DNA binding	N/A	N/A
*Afu5g12720*	Partial	ATP binding; ATPase activity, coupled to transmembrane movement of substances	transmembrane transport	integral component of membrane
*Afu6g00100*	Partial	nucleic acid binding	N/A	N/A
*Afu6g04480*	Partial	DNA binding; zinc ion binding	N/A	N/A
*Afu6g04490*	Partial	N/A	negative regulation of sexual sporulation resulting in formation of a cellular spore; positive regulation of asexual sporulation resulting in formation of a cellular spore	N/A
*Afu6g14630*	Partial	N/A	N/A	N/A
*Afu6g14640*	Complete	N/A	transmembrane transport	integral component of membrane
*Afu8g00342*	Partial	N/A	N/A	N/A
*Afu8g06132*	Partial	N/A	N/A	N/A
*Afu8g06140*	Complete	ATP binding; phosphorelay response regulator activity; phosphorelay sensor kinase activity; protein histidine kinase activity	peptidyl-histidine autophosphorylation; phosphorelay signal transduction system; regulation of transcription, DNA-templated	membrane
*Afu8g06150*	Complete	N/A	N/A	N/A
*Afu8g06160*	Complete	sequence-specific DNA binding; transcription factor activity, sequence-specific DNA binding	regulation of transcription, DNA-templated	nucleus
*Afu8g06165*	Complete	N/A	N/A	N/A

### Low levels of polymorphic copy number variation within *A*. *fumigatus* populations

**Fig 8:**
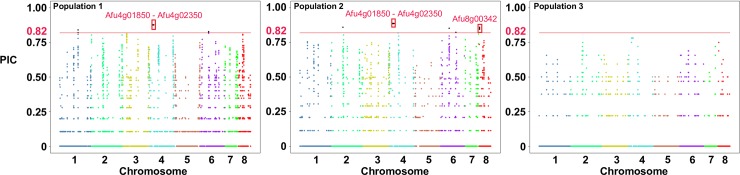
Highly polymorphic copy number variants within *A*. *fumigatus* populations. Manhattan plot of the Polymorphic Index Content (PIC) value (Y-axis) for each 500 bp non-overlapping window in the genome (x-axis) across populations 1 (A), 2 (B), and 3 (C). The horizontal red line represents the collective upper 99% percentile of PIC values across the 43 isolates. The loci outlined in red boxes represent the regions that overlapped entire genes.

We characterized patterns of CNV within populations by independently calculating PIC for each 500 bp window in populations 1, 2, and 3 (Fig 8). The average PIC values were 0.050, 0.036, and 0.016 for populations 1, 2, and 3, respectively. Population 3 harbors the lowest levels of intrapopulation CNV, which is in agreement with a previous report of low genetic variation [9]. We considered PIC values in the upper 99^th^ percentile of all populations as displaying significant levels of CNV within individual populations (PIC > 0.82). We identified 4 (278.5 kb), 5 (283.5 kb), and 0 significantly divergent CN variable loci within populations 1, 2, and 3. Three and four genes overlap significant PIC regions in populations 1, and 2 respectively, including three rDNA encoding genes. An additional gene (*Afu00342*) was identified in population 2, and neighbors the Pseurotin A encoding cluster [50, 55].

## Discussion

In this study, we identified and characterized CN variants on a genetically and geographically diverse collection of 71 *A*. *fumigatus* isolates. Our results reveal that, on average, 7.88% of the genome is CNV, among which absence and duplications account for 75.38% and 24.64%, respectively. Interestingly, the distribution of CN absences displayed a strong subtelomeric bias (Fig 3). This is consistent with previous research suggesting that the *A*. *fumigatus* subtelomeric regions are hypervariable [24, 48]. The subtelomeric bias of CNVs is also in line with results observed in *S*. *cerevisiae* [56, 57].

Enrichment analysis of genes overlapping absence revealed an interconnected set of GO terms associated with transposable elements (Table 1). Though the *A*. *fumigatus* genome is relatively compact, ~3% of the genome is composed of *Copia*, *Gypsy*, *I (LINE)*, and *Mariner* family transposable elements [58]. Transposable element CN varies at the population level in *Aspergillus* species [24, 59–61]. In rare cases, transposable element activity can lead to adaptive gene duplication, as in the *Tc1/mariner* induced duplication of alpha-amylase in *A*. *oryzae* [62]. The presence of transposable elements may promote CNV through retrotranspotion or nonallelic homologous recombination [63] and could account for some of the gene CNV between isolates.

The *A*. *fumigatus* genome contains 36 putative secondary metabolic gene clusters that encode a diverse set of compounds functioning in defense and communication [1, 47]. For example, the secondary metabolites gliotoxin, restrictocin, and fumagillin can induce host cell apoptosis, inhibit neutrophil-mediated hyphal damage, and cause epithelial cell damage and slowed ciliary beating, respectively [1]. We identified partial or entire gene absence in 16 secondary metabolic gene clusters in at least one isolate. These gene content polymorphisms could potentially affect the structure, expression, or transport of secondary metabolites [22]. For example, *Afu3g02570* encodes a nonribosomal peptide synthetases that acts as the backbone enzyme in a 21 gene secondary metabolic cluster [28]. This gene is absent in 59% of the isolates analyzed in this study. Additionally, we observed gene absence of the secondary metabolic cluster backbone enzymes *Afu1g01010* and *Afu2g17690* [55]. Conversely, one isolate (Afum IFM 62115) contained duplications of two entire secondary metabolic gene clusters, including a 22 gene cluster on chromosome 6 overlapping the fumisoquin cluster (*Afu6g03340*—*Afu6g03610*), and the fumagillin cluster on chromosome 8 (*Afu8g00370*—*Afu8g00570*) (Fig 5) [47]. Taken together, these results are consistent with previous reports of extensive genetic variation in *A*. *fumigatus* secondary metabolic clusters [22], and suggests that gene CNV in these regions could contribute to individual variation in secondary metabolite production, and pathogenicity.

Population differences in gene CN can be the result of natural selection favoring polymorphisms that are advantageous to a particular environment [19–21, 64–67]. Consistent with other species, the vast majority of CN variable loci were not stratified by population [21, 51, 68]. However, we identified 33 genes with highly differentiated CN profiles between populations 1, 2, and 3 (Fig 6 and Table 3). Many of these genes encode proteins that localized to or interact with the cell surface such as transporters, kinases, and hydrolases. For instance, *fhk3* (*Afu8g06140)* encodes a histidine kinase and is predominantly present at a CN of 1 in population 1, while entirely absent in populations 2 and 3 (Fig 6B). A knockout mutant of *fhk3* was not phenotypically distinct from the wild type strain, although only a limited number of environments and phenotypes were evaluated [69]. *Afu4g01070* is another gene overlapping a high V_ST_ region of the genome and encodes an acid phosphatase (Fig 7). Phosphate acquisition and storage is essential for the biosynthesis of nucleic acids, sugars, proteins, and lipids. In fungal pathogens, phosphate acquisition can also mediate resistance to alkaline pH, cation, oxidative, and nitrosative stress [70]. Populations 1 and 2 have a CN of 1, while seven of the eight isolates in population 3 contain an absence that accounts for nearly half of the gene length. Previous molecular characterization of *PHO80* suggest that *Afu4g01070* is likely involved in the acquisition of inorganic phosphate [71]. Consistent with studies in *Cyptococcus gattii* and *S*. *cerevisiae* [19, 21], our results suggest that CNV genes often localize to the cell surface and could be the result of sensing and responding to population-specific environmental factors.

Alvoelar macrophages kill swollen conidia with reactive oxygen species (ROS) [1]. To survive this immune response, *A*. *fumigatus* has evolved several strategies to counteract ROS, including the ability to produce of a variety of oxidation-reduction enzymes. We identified two adjacent genes with predicted functions in oxidation-reduction (*Afu3g00100 and Afu3g00110*) that displayed divergent CN patterns (Fig 7 and Table 3). Both genes were predominantly present at a CN of 1 in population 1 and entirely absent in populations 2 and 3. Lastly, we observed a CN divergent region that overlaps *Afu6g04490*. This regulatory gene is involved in sporulation and asexual development (Table 3). This gene was present at a CN of 1 in population 1, and ranged between 1 and 6 in populations 2 and 3. The *Aspergillus nidulans* ortholog (*osaA*) functions as a transcription factor that regulates sexual and asexual development [72]. Multiple copies of *osaA* in the *A*. *nidulans* genome leads to proliferation of vegetative cells while deletion of *osaA* results in heightened sexual fruiting and reduces asexual development [72]. In *Fusarium oxysporum* the otholog *Sge1* is also confirmed as a transcription factor and is essential for pathogenicity in tomato [73].

CNV is an often overlooked source of genetic variation [74]. We have conducted the first population genomic characterization of *A*. *fumigatus* CNV to better understand their abundance, localization, and potential functional associations. Further molecular and experimental studies are warranted to assess the functional role of CNVs in *A*. *fumigatus*. More broadly, to fully grasp the influence of genetic variation on phenotype, there is a need for the *A*. *fumigatus* community to combine comparative, population, and quantitative genomics [9, 23, 54] with functional genomics [75–78], proteomics [79–82], high-throughput phenotyping [83–86], and molecular strategies such as RNA interference [87, 88] and CRISPR/Cas9 [89].

## Supporting information

S1 Fig Phylogenetic network generated in Splitstree4 of the 71 *A*. *fumigatus* isolates [35]. The scale bar represents the proportion of nucleotide sites at which two sequences that were being compared were different. Isolates colored with red, blue, and yellow correspond to structure populations 1, 2, and 3.
(PDF)

S1 Table Metadata for all analyzed *A*. *fumigatus* isolates. (XLSX)

S1 File Genome-wide copy number estimates for each non-overlapping 500 bp window for all *A*. *fumigatus* isolates. (CSV)
