# Supplementary figures and images for: A population genomic characterization of copy number variation in the opportunistic fungal pathogen Aspergillus fumigatus
Source: PLoS One. 2018 Aug 2;13(8):e0201611. doi: 10.1371/journal.pone.0201611 (PMC6072042; doi:10.1371/journal.pone.0201611)

## S1 Figure

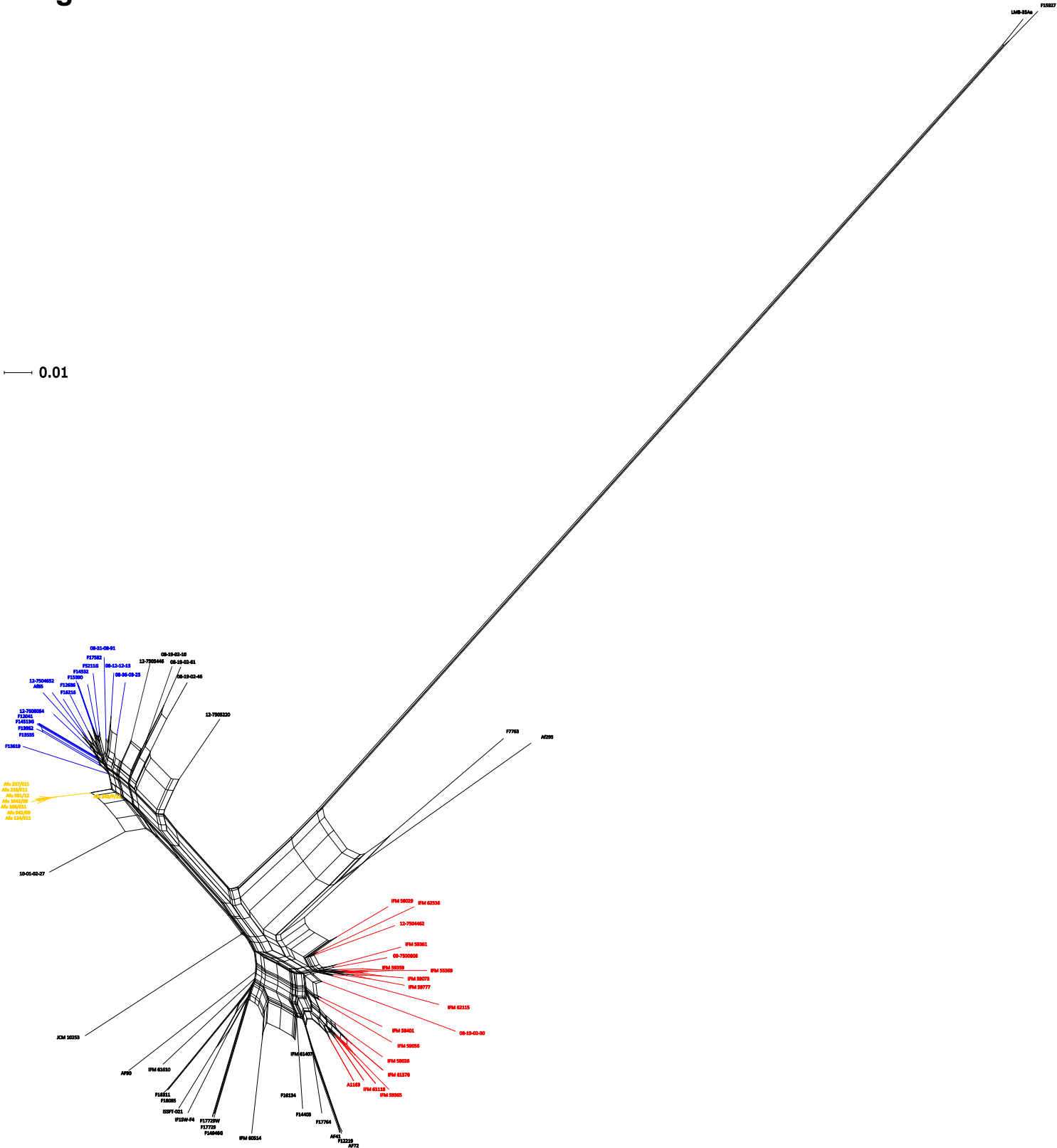

Supplement: S1 Fig — The scale bar represents the proportion of nucleotide sites at which two sequences that were being compared were different. Isolates colored with red, blue, and yellow correspond to structure populations 1, 2, and 3. (PDF) [file pone.0201611.s001.pdf]
